# Ischemic Preconditioning Enhances Aerobic Adaptations to Sprint-Interval Training in Athletes Without Altering Systemic Hypoxic Signaling and Immune Function

**DOI:** 10.3389/fspor.2020.00041

**Published:** 2020-04-28

**Authors:** Pénélope Paradis-Deschênes, Denis R. Joanisse, Pascale Mauriège, François Billaut

**Affiliations:** ^1^Département de kinésiologie, Université Laval, Québec, QC, Canada; ^2^Institut Universitaire de Cardiologie et de Pneumologie de Québec, Québec, QC, Canada

**Keywords:** angiogenesis, blood-flow restriction, HIIT, hypoxia, NIRS, peripheral adaptation

## Abstract

Optimizing traditional training methods to elicit greater adaptations is paramount for athletes. Ischemic preconditioning (IPC) can improve maximal exercise capacity and up-regulate signaling pathways involved in physiological training adaptations. However, data on the chronic use of IPC are scarce and its impact on high-intensity training is still unknown. We investigated the benefits of adding IPC to sprint-interval training (SIT) on performance and physiological adaptations of endurance athletes. In a randomized controlled trial, athletes included eight SIT sessions in their training routine for 4 weeks, preceded by IPC (3 × 5 min ischemia/5 min reperfusion cycles at 220 mmHg, *n* = 11) or a placebo (20 mmHg, *n* = 9). Athletes were tested pre-, mid-, and post-training on a 30 s Wingate test, 5-km time trial (TT), and maximal incremental step test. Arterial O_2_ saturation, heart rate, rate of perceived exertion, and quadriceps muscle oxygenation changes in total hemoglobin (Δ[THb]), deoxyhemoglobin (Δ[HHb]), and tissue saturation index (ΔTSI) were measured during exercise. Blood samples were taken pre- and post-training to determine blood markers of hypoxic response, lipid-lipoprotein profile, and immune function. Differences within and between groups were analyzed using Cohen's effect size (ES). Compared to PLA, IPC improved time to complete the TT (Mid vs. Post: −1.6%, Cohen's ES ± 90% confidence limits −0.24, −0.40;−0.07) and increased power output (Mid vs. Post: 4.0%, ES 0.20, 0.06;0.35), Δ[THb] (Mid vs. Post: 73.6%, ES 0.70, −0.15;1.54, Pre vs. Post: 68.5%, ES 0.69, −0.05;1.43), Δ[HHb] (Pre vs. Post: 12.7%, ES 0.24, −0.11;0.59) and heart rate (Pre vs. Post: 1.4%, ES 0.21, −0.13;0.55, Mid vs. Post: 1.6%, ES 0.25, −0.09;0.60). IPC also attenuated the fatigue index in the Wingate test (Mid vs. Post: −8.4%, ES −0.37, −0.79;0.05). VO_2_peak and maximal aerobic power remained unchanged in both groups. Changes in blood markers of the hypoxic response, vasodilation, and angiogenesis remained within the normal clinical range in both groups. We concluded that IPC combined with SIT induces greater adaptations in cycling endurance performance that may be related to muscle perfusion and metabolic changes. The absence of elevated markers of immune function suggests that chronic IPC is devoid of deleterious effects in athletes, and is thus a safe and potent ergogenic tool.

## Introduction

One of the great challenges for coaches and sports scientists is to identify ergogenic strategies to optimize training adaptations and performance in athletes for whom adaptations are harder to elicit (Laursen and Jenkins, 2002; Taylor et al., 2016a). Indeed, there is a scope to find new approaches to enhance traditional training methods, and ischemic preconditioning (IPC) seems promising to achieve this goal. This non-invasive technique, involving repeated episodes of muscle ischemia followed by reperfusion at rest, induces transient peripheral hypoxia and can acutely improve maximal exercise capacity (Cruz et al., 2016; Salvador et al., 2016). For example, IPC improved mean power output during a 60 s cycling sprint, with a greater increase at exercise onset (Cruz et al., 2016), and time-trial (TT) performance in cyclists (Paradis-Deschênes et al., 2018; Wiggins et al., 2018). However, the precise physiological responses and mechanisms associated with these enhancements are still equivocal. Among the possibilities, IPC could impact performance by improving local vasodilation, blood flow, and ultimately O_2_ uptake kinetics during maximal efforts (Enko et al., 2011; Bailey et al., 2012a; Paradis-Deschênes et al., 2016; Kilding et al., 2018). Ergogenic molecular and vascular adaptations within conduit arteries and capillary beds have also been suggested. Shear stress and local tissue hypoxia induced by the maneuver increased nitric oxide (NO) levels, a potent vasodilator (Lochner et al., 2002), activated vascular endothelial growth-factor (VEGF-α) gene expression and up-regulated hypoxia inducible factor-1α (HIF-1α) (Fukumura et al., 2001; Albrecht et al., 2013; Heusch et al., 2015). Thus, IPC could readily increase exercise tolerance, thereby allowing a greater training load and subsequent physiological adaptations, notably by enhancing tissue hypoxia-mediated cell signaling.

The literature on the chronic effects of IPC on performance is scarce. Repeated exposure to IPC for 7 to 9 days did not change (Banks et al., 2016) or increase maximal O_2_ consumption (VO_2_max) (Lindsay et al., 2017). Improvements in maximal aerobic power (MAP) and anaerobic capacity (i.e. increase in peak power output, mean power output, and fatigue index) during repeated Wingate tests (Lindsay et al., 2017) have also been reported. However, in athletes, the application of IPC alone would likely be insufficient to induce such improvements and, in fact, IPC applied once or twice a day for 7 consecutive days failed to improve 4-km TT performance in endurance cyclists (Lindsay et al., 2018). To date, only one study used IPC prior to high-intensity training sessions, but authors reported that neither training alone nor training preceded by IPC improved VO_2_max and 1-km TT running performance in distance runners after 8 weeks (Slysz and Burr, 2019). This is surprising considering the previously mentioned acute effects of IPC, and may partly be explained by: (1) the absence of effects from the 8 weeks of intensified training *per se* (comprising a majority of low intensity running interspersed with tempo and 1-km pace runs), and (2) the use of unilateral occlusions, which are suspected to be an insufficient stimulus to derive benefits from IPC (Kraus et al., 2014; Cocking et al., 2018). Moreover, recent studies on blood-flow restriction, another modality that targets metabolic functions by impacting blood flow, also highlight the potency of such hypoxic strategies to augment metabolic stimulus and induce greater adaptive responses (Taylor et al., 2016a; Mitchell et al., 2019). For example, Taylor et al. combined blood-flow restriction and sprint-interval training (SIT) and reported an increase in VO_2_max and a greater expression of the hypoxic growth factor HIF-1α 3 h after a single session, which was not the case after SIT alone (Taylor et al., 2016a).

SIT is typically characterized by fewer than 12 repeated supra-maximal or “all-out” efforts lasting 10 to 30 s that are interspersed by ~2 to 5 min of passive recovery, and is commonly used to induce aerobic and anaerobic adaptations (Sloth et al., 2013). However, although 2 to 8 weeks of SIT can increase VO_2_max and performance during TT and Wingate anaerobic tests (Burgomaster et al., 2006; Hazell et al., 2010; Sloth et al., 2013), its efficacy on trained individuals is still equivocal. Interestingly, some characteristics ascribed to this type of training could benefit from IPC. Indeed, SIT intensity, particularly the peak power generated during the first seconds of exercise and the subsequent repeated metabolic stress, has been suggested to be of particular relevance to adaptations (Hazell et al., 2010; Skelly and Gillen, 2018). The increase in motor unit recruitment with all-out efforts is likely responsible for the enhancement of oxidative capacity and metabolic profile of type II fibers (Bailey et al., 2009), while metabolic perturbations promote the increase of signaling processes of different transcription factors for angiogenesis and mitochondrial biogenesis (Daussin et al., 2008; Psilander et al., 2010; Cocks et al., 2013), including HIF-1α and VEGF-α (Lee et al., 2004; Taylor et al., 2016b). Thus, the addition of IPC could enhance adaptations to SIT by increasing early exercise power output, further stimulating adaptive signaling pathways.

Therefore, the aim of the current project was to determine the effect of the combination of IPC and SIT on performance and physiological adaptations. We hypothesized that IPC and SIT, compared to SIT alone, would induce greater improvement in VO_2_peak, 30 s Wingate test, and 5-km TT performance, and that the TT enhancement would be concomitant to muscle oxygenation adaptations. We also hypothesized that blood markers of angiogenesis and vasodilation would be elevated after 4 weeks of training in the IPC group.

## Materials and Methods

### Ethics Approval

The study was approved by the Ethics Committee of University Laval, and adhered to the principles established in the Declaration of Helsinki. Participants provided written informed consent after being informed of experimental procedures, associated risks, and potential benefits.

### Participants

Twenty seven participants were recruited, seven dropped out due to external commitments or injuries unrelated to the study protocol, and 20 completed the study. Subjects trained on average 6.5 ± 0.5 h/week in an endurance sport (e.g., cycling, running, swimming, etc.) at the time of the study, had a training history longer than 2 years in their respective sport, and a minimal cycling experience. All participants were non-smokers, free of health problems, and did not use any medication or any other tobacco/nicotine products.

### Study Design

Participants visited the laboratory for a total of 16 sessions, including eight training sessions spread over 4 weeks and 8 testing days for pre- (4), mid- (1), and post-training (3) evaluations. Pre-training evaluations were divided as follows and described below (see *experimental protocol)*: (1) maximal incremental step test, (2) familiarization, (3) body fat and fasted blood samples, and 4) 30 s Wingate and 5-km TT tests. Using a between-groups design, participants were pair-matched based on age, VO_2_peak, MAP, and TT performance, as well as on their relative peak power (PPO) and mean power output (MPO) obtained during the Wingate test, and then randomly assigned to IPC or PLA (dice roll) to obtain equivalent groups for every pre-testing variable. Both groups had an identical training intervention (see *training intervention)*. Mid-training evaluation included a Wingate Test and a 5-km TT and was executed 2 to 4 days after the fourth training session. Finally, all testing procedures were repeated post-training, but in a different order: (1) body fat and fasted blood samples, (2) 30 s Wingate and 5-km TT tests, and (3) maximal incremental step test.

All sessions were performed at the same time of the day to avoid potentially confounding circadian rhythm effects and were separated by a minimum of 2 days to avoid fatigue. Temperature (21.5 ± 0.1°C, mean ± SE) and humidity (29.9 ± 1.0%) were kept constant. Prior to each testing and training day, vigorous exercise was avoided for 48 h and alcohol and caffeine were refrained from for 24 h. During all exercise protocols and training sessions, participants were instructed to remain seated and were strongly verbally encouraged. The handlebars and seat settings of each device (Lode, Velotron, Keiser) were individualized and replicated throughout the study. Participants were also asked to replicate non-prescribed training throughout the entire study and record perceived exertion and duration of every physical activity in a training log book (Haddad et al., 2017).

### Experimental Protocol

#### Maximal Incremental Step Test

This session was preceded by measurement of resting heart rate (HR) and blood pressure (inclusion criteria <100 beats per minute and <140/90 mmHg) in a seated position and baseline characteristics: body height, body mass, thigh skinfold thicknesses (IPC: 5.3 ± 0.7 mm; PLA: 6.7 ± 0.6 mm), and thigh circumference (IPC: 55.6 ± 1.2 cm; PLA: 54.9 ± 1.2 cm). The participant was then positioned on an electromagnetically-braked cycle ergometer (Excalibur Sport, Lode, the Netherlands) for a 2 min baseline in a seated position and a 5 min warm-up at 100 W before the maximal step test (30 W per minute until volitional exhaustion). Expired gases were analyzed breath-by-breath throughout the test (Breezesuite, MedGraphics Corp., Minnesota, Saint Paul, USA) to assess the VO_2_, carbon dioxide production (VCO_2_), and respiratory exchange ratio. VO_2_peak was taken as the highest 20 s average recorded during the test, and VCO_2_peak, peak respiratory exchange ratio, peak respiratory rate, and MAP were also averaged at the time. The respiratory compensation point was determined by two researchers by identifying an increase in ventilatory equivalents for O_2_ and CO_2_ concomitant to a decrease in end-tidal partial pressure of CO_2_ (Wasserman et al., 1999) plotted against VO_2_. Values at the respiratory compensation point are reported in absolute and relative values (i.e., compared to those at maximal exercise such as %MAP, %VO_2_peak, %VCO_2_peak). Participants were asked to replicate the same diet at least 2 h before each maximal incremental step test.

#### Familiarization

This session was similar to testing procedures described below, but with a shorter rest between the 30 s Wingate Test and the 5-km TT (15 vs. 30 min), and without any analysis devices.

#### Body Fat and Fasted Blood Samples

Participants fasted for 12 h before these sessions which occurred 2 days after the familiarization and the last SIT session, for pre- and post-training assessment, respectively. During this session, the body fat percentage was measured by bioelectrical impedance (Tanita TBF-310; Tanita Corp. of America Inc., Arlington Heights, IL) and blood samples (~24 mL) were collected from the antecubital vein in a semi-recumbent position by a registered nurse (see *blood measurements* below).

#### 30 s Wingate and 5-km TT Tests

For pre-, mid-, and post-training evaluations, the steps were always as follows: 5 min rest in a supine position (NIRS baseline recording), 10 min standardized warm-up, 2 min rest in a seated position, 30 s Wingate test, 5 min cool down, 30 min rest in a semi-recumbent position, 10 min standardized self-paced warm-up, 2 min rest in a seated position, 5-km TT, and 5 min cool down.

For the standardized warm-up preceding the Wingate test, participants were instructed to choose their preferred cadence at a low resistance for 6 min. This was followed by three 5 s sprints at a greater resistance (85–95–100%), interspersed by 15 s active recovery. After these sprints, participants completed the warm-up at their chosen pace and resistance. For the standardized and self-paced warm-up (preceding TT), power output, speed, and gear were continuously noted by the experimenter and strictly reproduced thereafter.

The 30 s Wingate test and the 5-km TT were executed on a computer-controlled electrically braked cycle ergometer (Velotron Elite, RacerMate, Seattle, WA, USA). For the Wingate test, pedaling rate was gradually increased in order to reach 100 W during 20 s and followed by a 3 s acceleration phase to attain a peak power as fast as possible. During the 30 s of maximal exercise phase of the Wingate test, a resistance equivalent to 7.5% of each participant's body mass was computer-controlled (Wingate Software Version 1.11, Lode BV). For the TT, participants were instructed to complete the 5 km as quickly as possible, with the distance traveled as the only available information. Measurements during these tests are described in the following section. To control for diet and activity patterns prior to these sessions, participants were asked to record and replicate their dietary intake and physical activity respectively for 24 and 72 h before testing.

### Training Intervention

All SIT sessions were performed at the training center of Université Laval on Keiser M3+ cycle ergometers (Keiser corporation, Fresno, CA), preceded by IPC or PLA procedures, and supervised by an investigator. Training volume was increased progressively weekly with two sessions of four, five, six, and seven 30 s all-out efforts separated by 4 min and 30 s over 4 weeks. The warm-up was identical as the one performed during the Wingate tests. All parameters (indoor cycle model, resistance, handlebars, and seat settings) were replicated for all training sessions, which always proceeded as follows: IPC or PLA application, 10 min standardized warm-up, 2 min seated rest, 30 s all-out efforts, and 5 min cool-down. The 30 s all-out effort was preceded by an acceleration of 15 s including a 5 s countdown to increase speed and resistance. After the all-out effort, participants had a 15 s period of cycling without any resistance at a chosen pace before the 4 min passive rest. During each sprint, the peak and the minimum power were noted by the investigator and participants gave their RPE scores to calculate session RPE (RPE scores × duration).

### Ischemic Preconditioning

Non-elastic nylon blood pressure cuffs (WelchAllyn, Skaneateles Falls, NY, USA, width: 21 cm) were positioned around each upper thigh under the gluteal line and rapidly inflated to 220 mmHg (IPC) or 20 mmHg (PLA) for 5 min to prevent arterial inflow, three times per limb, alternatively, with each compression episode separated by 5 min of reperfusion (cuff release). This protocol has previously been shown to alter physiological responses and enhance performance (Bailey et al., 2012a; Paradis-Deschênes et al., 2018), and to completely occlude vascular arterial inflow (Sabino-Carvalho et al., 2016). To minimize any placebo effect, participants were told that the purpose of the study was to compare the impact of two different cuff pressures that could both alter training positively, with venous or arterial effects, according to the pressure used. The participants in the IPC group were familiarized with the pressure before the first training session.

### Instrumentation and Measurements

#### Arterial O_2_ Saturation (SpO_2_) and heart rate (HR)

S_p_O_2_ and HR were recorded at the end of each step of the maximal incremental step test and every 250 m during TTs from a pulse oximeter (Nellcor Bedside, Nellcor Inc. Hayward, CA) with an adhesive forehead sensor secured with a headband. This technique has been shown to be in good agreement with hemoglobin O_2_ saturation based on arterial blood analysis over the 70–100% range (Romer et al., 2007). The S_p_O_2_ measured at the forehead is also highly correlated with S_a_O_2_ measured by direct arterial blood measurements (*R*^2^ = 0.90, *P* < 0.0001) and has significantly lower bias and greater precision for S_p_O_2_ (0.3 ± 1.5%) and HR (1.8 ± 5.5%) than finger probes in athletes (Yamaya et al., 2002).

#### Near-Infrared Spectroscopy (NIRS)

A portable spatially-resolved, dual wavelength NIRS apparatus (PortaMon, Artinis Medical Systems BV, The Netherlands) was installed on the distal part of the right vastus lateralis muscle (~15 cm above the proximal border of the patella), parallel to muscle fibers, to quantify changes in the absorption of near-infrared light by oxy-hemoglobin (HbO_2_) and deoxy-hemoglobin (HHb). The skinfold thickness was measured at the site of the application of the NIRS using a Harpenden skinfold caliper (British Indicators Ltd, West Sussex, Great Britain) during the first session, and was less than half the distance between the emitter and the detector (i.e., 20 mm). This thickness allows for adequate penetration of near-infrared light into muscle tissue for valid measurements (Mccully and Hamaoka, 2000). The device was packed in transparent plastic wrap to protect it from sweat and fixed with tape. Black bandages were used to cover the device from interfering background light. The position of the apparatus on the thigh was marked with an indelible pen for repositioning during the same block of tests and a picture was taken for a better replacement between different blocks (i.e., from pre- to post-training). The pressure cuff used to induce IPC or PLA was positioned above the NIRS device and did not affect the placement of the device.

A modified form of the Beer-Lambert law, using two continuous wavelengths (760 and 850 nm) and a differential optical path length factor of 4.95 was used to calculate micromolar changes in tissue [HbO_2_], [HHb] and total hemoglobin ([THb] = [HbO_2_] + [HHb]). Changes in tissue saturation index (TSI = [HbO_2_]/[THb]) were also used as an index of tissue oxygenation since it reflects the dynamic balance between O_2_ supply and consumption in the tissue microcirculation (Van Beekvelt et al., 2001; Ferrari et al., 2004). This parameter is independent of near-infrared photon path length in tissue. Before each test, 1 min of baseline values was analyzed, once the signal was stabilized, to express the magnitude of change during exercise relative to the baseline values (Δ[HHb], Δ[THb], ΔTSI). Thus, all NIRS variables are expressed as the differences between exercise and rest values (Δ). Δ[HHb] was taken as an index of muscle O_2_ extraction (Van Beekvelt et al., 2002), and Δ[THb] as a change in regional blood volume (Van Beekvelt et al., 2001). NIRS data were acquired continuously at 10 Hz for every testing session. A 10th order zero-lag low-pass Butterworth filter was applied to smooth NIRS signal (Paradis-Deschênes et al., 2018). Data were averaged over 10 s at the end of each step of the maximal cycling test and leading up to every 250 m of the TT. For the Wingate test, data were averaged over 2 s at the end of the test and the 30 s period, for the peak and the mean values, respectively.

#### Power Output

The power output was continuously recorded from the Lode (maximal incremental step test) and the Velotron ergometer (30 s Wingate test and 5-km TT). For the 30 s Wingate test, MPO was averaged along 30 s and PPO was the highest power output over 1 s. The fatigue index was also calculated (FI = [PPO—lowest 1 s power output]/PPO × 100). For the 5-km TT, power output was averaged over a period of 10 s leading up to every 250 m.

#### Ratings of Perceived Exertion (RPE)

The RPE scores were recorded at the end of every step of the VO_2_peak test and every 500 m of the TT using the Borg 10-point scale to assess subjective perceived exertion.

#### Blood Measurements

Blood was collected in four tubes from an antecubital vein in the morning after at least a 12 h overnight fast and a 15 min rest period. One tube of 6 mL of plasma was kept at room temperature and three tubes of 6 mL of serum were placed on ice during blood collection and centrifuged at 3,000 rpm for 10 min for the following analysis. Twelve mL of serum were divided in four aliquots and stored at −80°C until the end of the project for HIF-1α, VEGF, NO, and free-fatty acid analyses. HIF-1α and VEGF-α levels were determined by high-sensitivity enzyme-linked immuno-absorbent assays (ELISA) (Invitrogen, Thermo Fisher Scientific, Ontario) while total NO was assessed with a colorimetric assay kit (Invitrogen, Thermo Fisher Scientific, Ontario). The reproducibility of the HIF-1α kit displayed intra-assay and inter-assay CV values lower than 10%, irrespective of the concentration. Concerning the VEGF-α kit, CV values for intra-assay precision were 5.5 and 4.9% while those for inter-assay precision were 9.3 and 6.5%, for low and high VEGF-α levels, respectively. For the NO kit, intra-assay precision showed CV values of 5.3 and 1.2% while inter-assay precision presented CV values of 6.9 and 3.3%, for low and high nitrate concentrations, respectively.

The serum sample (6 mL) and the fourth tube of plasma of 6-mL were sent immediately after blood sampling and analyzed within 2 h at the Quebec Heart and Lung Institute. Plasma was analyzed using the LH 780 (Beckman Coulter) for a complete blood profile (including blood count, hemoglobin, hematocrit, etc.). Serum was analyzed using standardized laboratory procedures on a Dimension Vista 1500 Intelligent Lab System (Siemens) to determine ferritin concentration by chemiluminescent ELISA and the lipid-lipoprotein profile. Fasting total cholesterol, triglyceride, and high-density lipoprotein (HDL)-cholesterol levels were determined by colorimetry. Fasting low-density lipoprotein (LDL)-cholesterol concentrations were estimated using the Friedewald equation (Friedewald et al., 1972). Free-fatty acid levels were enzymatically measured by a colorimetric method (Wako Chemicals, Ontario). Fasting glucose was analyzed using a Dimension Vista 1500 Intelligent Lab System (Siemens) by photometry and the insulin levels using ADVIA Centaur XPT (Siemens) by immunoassay. All measurements were performed in duplicate, and then averaged.

### Statistical Analysis

We evaluated the magnitudes of difference within groups from pre- and mid-training to post-training for all variables as well as the percentage difference between change in IPC and PLA during the first (0 to 2.5 km) and the second (2.6 to 5 km) half of the TT and the entire TT. Practical significance was evaluated using Cohen's effect sizes (ES) ± 90% confidence limits, and compared to the smallest worthwhile change that was calculated as the standardized mean difference of 0.2 between-subject standard deviations (Batterham and Hopkins, 2006; Hopkins et al., 2009). All variables were log-transformed before analysis (Hopkins et al., 2009), except for Δ[THb] and HIF-1α concentration, and raw data are reported as mean ± standard error (SE) for clarity. Standardized effects were classified as small (0.2–0.49), moderate (0.5–0.79), or large (≥0.8) (Hopkins et al., 2009). Using mechanistic inferences, qualitative probabilistic terms for benefit were assigned to each effect for mechanical, NIRS, cardiorespiratory, blood markers, and perceptual variables using the following scale: 50 to 75%, possibly; 75 to 95%, likely; 95 to 99.5%, very likely; >99.5%, almost certainly. The effect of IPC was deemed “unclear” if chances of having better/greater and poorer/lower changes in performance and physiological variables were both >5% (Batterham and Hopkins, 2006; Hopkins et al., 2009). Pearson correlations were calculated to assess associations between physiological changes and performance improvements. Correlation coefficients of > 0.1, > 0.3, > 0.5, and > 0.7 were considered small, moderate, large, and very large (Hopkins et al., 2009).

## Results

All participants completed all assigned sessions and tolerated the IPC procedure without complications. The values of two participants in the IPC group for the mid-training sessions (two Wingate tests and one TT) were not included because of food indigestion and technical problems with the Velotron ergometer. Baseline characteristics between IPC (age 31.5 ± 3.0 yr; body mass 76.3 ± 2.9 kg; height 1.80 ± 0.02 m; body fat 12.0 ± 2.0 %, *n* = 11) and PLA (28.1 ± 2.5 yr; 74.3 ± 3.4 kg; 1.76 ± 0.03 m; 10.2 ± 1.6 %, *n* = 9) groups were not significantly different and were not altered by the intervention. Prior to training, there was no difference in any physiological and performance variables between groups (VO_2_peak, MAP, TT cycling time, PPO, and MPO).

### 30 s Wingate test

#### Performance Parameters

Table 1 displays mechanical and muscle oxygenation variables during the Wingate tests. Both groups increased power output over the course of the training. The only between-group differences were that the increase in PPO relative to body mass from mid- to post-training was possibly lower in IPC. However, IPC likely decreased the fatigue index from mid- to post-training compared to PLA.

**Table 1:** Mechanical and near-infrared spectroscopy variables during the 30 s Wingate test after IPC and PLA at pre-, mid- and post-training.

	**IPC**			**PLA**			**ΔIPC vs**. **ΔPLA**	
	**PRE**	**MID**	**POST**	**PRE**	**MID**	**POST**	**MID vs. POST**	**PRE vs. POST**
	**Mean** **±** **SE**			**Mean** **±** **SE**			**%D (ES) CL**	
MPO (W)	751 ± 35	752 ± 34	759 ± 32	705 ± 34	709 ± 36	718 ± 32	−0.4% (−0.02) −0.22;0.17	−0.6% (−0.04) −0.23;0.16
MPO (W/kg)	9.9 ± 0.4	9.9 ± 0.4	10.0 ± 0.3	9.5 ± 0.3	9.5 ± 0.3	9.7 ± 0.3	−0.4% (−0.03) −0.29;0.23	−0.6% (−0.05) −0.30;0.21
PPO (W)	1078 ± 63	1126 ± 75	1131 ± 83	1008 ± 28	1053 ± 46	1094 ± 62^a,b^	−3.3% (−0.19) −0.53;0.16	−3.3% (−0.19) −0.66;0.29
PPO (W/kg)	14.1 ± 0.6	14.7 ± 0.7^a^	14.8 ± 0.8^a^	13.7 ± 0.6	14.2 ± 0.5	14.8 ± 0.6^a,b^	**−3.3% (−0.23)** **−0.64;0.19**	−3.3% (−0.23) −0.80;0.35
Fatigue index (%)	51.7 ± 2.6	54.9 ± 4.4	52.3 ± 5.2^b^	53.5 ± 3.7	54.4 ± 3.5	56.0 ± 3.8	**−8.4% (−0.37)** **−0.79;0.05**	−6.9% (−0.30) −1.01;0.40
Δ[HHb]mean (μM)	18.0 ± 2.4	18.6 ± 2.5	20.4 ± 3.1	14.5 ± 1.4	14.7 ± 1.6	14.6 ± 1.5	7.1% (0.13) −0.18;0.44	**13.9% (0.25)** **−0.12;0.62**
Δ[HHb]peak (μM)	19.6 ± 2.5	20.8 ± 2.7	22.5 ± 3.1^a^	15.7 ± 1.8	16.2 ± 1.9	15.8 ± 1.7	8.9% (0.17) −0.14;0.47	**15.3% (0.28)** **−0.09;0.64**
Δ[THb]mean (μM)	8.13 ± 2.6	6.73 ± 2.6	9.33 ± 2.7	6.46 ± 0.9	5.14 ± 1.1	4.03 ± 2.4^a^	**43.3% (0.42)** **−0.06;0.91**	**36.2% (0.38)** **−0.21;0.97**
Δ[THb]peak (μM)	11.6 ± 2.9	10.4 ± 2.8	11.5 ± 2.9	9.82 ± 1.2	7.66 ± 1.6	7.05 ± 2.5^a^	**20.8% (0.28)** **−0.10;0.66**	**28.4% (0.40)** **−0.16;0.96**
ΔTSI mean (%)	−32.7 ± 2.5	−30.7 ± 2.8	−35.6 ± 5.5	−32.1 ± 3.3	−33.2 ± 3.8	−39.1 ± 4.0^a^	−11.6% (−0.33) −1.41;0.76	**−17.4% (−0.51)** **−1.21;0.20**
ΔTSI peak (%)	−31.2 ± 2.4	−29.6 ± 2.5	−35.7 ± 5.1	−29.4 ± 3.2	−30.2 ± 3.8	−37.5 ± 3.6^a^	−17.7% (−0.42) −1.52;0.69	**−16.4% (−0.38)** **−0.97;0.20**

*CL, confidence limits; %D, percentage difference between changes in IPC and PLA; ES, effect size; Δ[HHb], change in deoxy-hemoglobin; MPO, mean power output; PPO, peak power output; Δ[THb], change in total hemoglobin; ΔTSI, change in tissue saturation index*.

a,b *clear changes within each group in post- compared to pre- and mid-training, respectively*.

*Differences between changes in IPC and PLA are indicated in bold*.

#### Muscle Oxygenation Variables

From pre- to post-training, IPC maintained the index of muscle blood volume (Δ[THb]_mean_ and Δ[THb]_peak_) whereas it clearly declined in PLA (Table 1). Similar modifications were observed from mid- to post-training, which yielded a clear between-group difference in these parameters. These changes were concomitant to those in the Wingate fatigue index in favor of IPC. There were also possible differences between IPC and PLA from pre- to post-training in Δ[HHb]_mean_, Δ[HHb]_peak_, ΔTSI_mean_, and ΔTSI_peak_. Moreover, from pre- to post-training, the change in PPO was correlated with Δ[HHb]_mean_ (*r* = 0.83) and Δ[HHb]_peak_ (*r* = 0.75) in IPC group, and with ΔTSI_mean_ (*r* = 0.79) and ΔTSI_peak_ (*r* = 0.78) in PLA group.

### 5-km Time Trial

#### Performance Parameters

**Figure 1:**
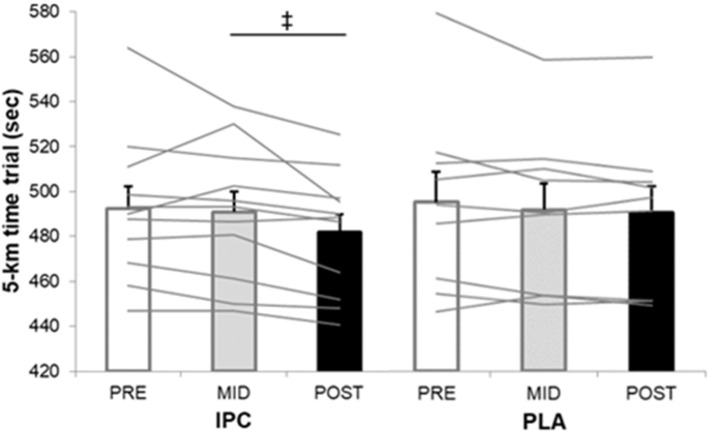
Average and individual completion times of the 5-km time trial at pre-, mid-, and post-training in IPC and PLA. IPC improved TT performance post- compared to pre- (↑2.1%, ES 0.32, 0.14;0.51) and mid- (↑1.8%, ES 0.28, 0.12; 0.44). (‡), clear post- compared to mid- between-group (IPC –PLA) differences (↑1.6%, ES 0.24, −0.07; 0.40). Values are mean ± SE.

Average and individual completion times of the TT are displayed in Figure 1. The calculated smallest worthwhile change for TT time in IPC and PLA group, respectively, equated to 5.94 s and 7.29 s from pre- to post-training, and to 5.74 and 6.80 s from mid- to post-training. The completion time in post- was likely faster in IPC compared to both pre- (−10.6 s, −2.1%, ES 0.32) and mid-training (−9.1 s, −1.8%, ES 0.28). Changes in PLA were trivial with improvements of 4.4 s from pre- to post-training. When comparing groups, IPC produced possible faster times from mid- to post-training (−8.0 s, −1.6%, ES 0.24) compared to PLA.

**Figure 2:**
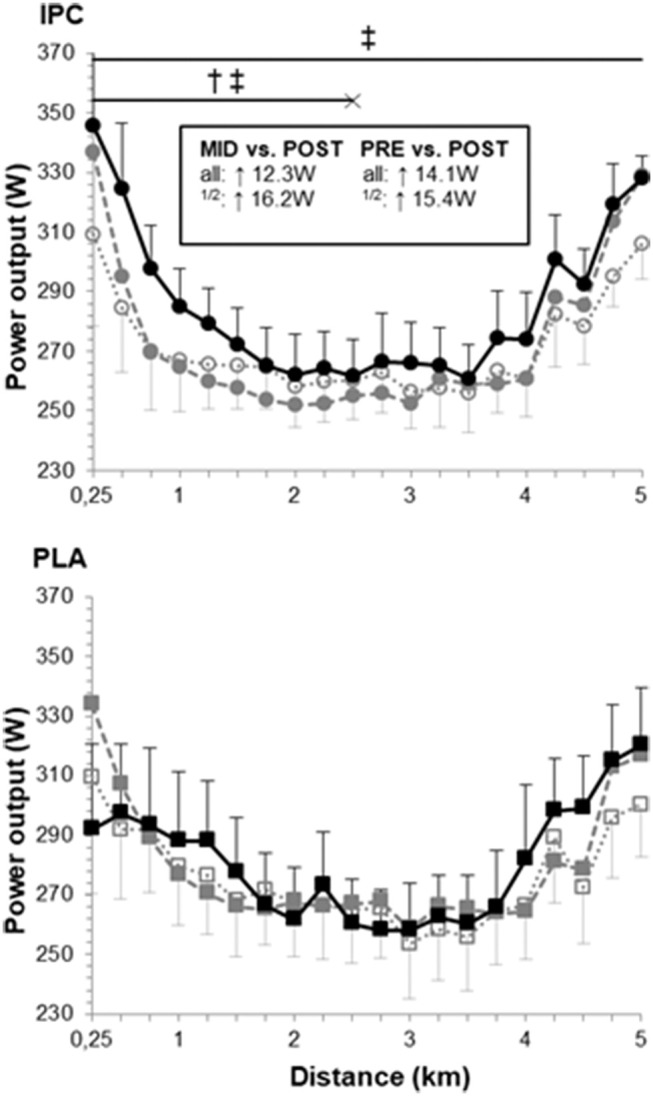
Power output profile during the 5-km time trial at pre- (open symbols), mid- (gray symbols) and post-training (black symbols) in IPC and PLA. Power outputs were as follows: pre- (IPC: 271 ± 13 W; PLA: 276 ± 18 W), mid- (IPC: 273 ± 13 W; PLA: 279 ± 17 W), and post-training (IPC: 285 ± 13 W; PLA: 281 ± 17 W). Within-group difference between time points are indicated in the square box for the first half (1/2) and the entire TT (all). (†) and (‡) indicate clear differences between-groups (IPC vs. PLA) at post- compared to pre- and mid-, respectively. Values are mean ± SE.

The power output profiles during the TT are displayed in Figure 2. IPC likely increased overall power output in post- compared with pre- (5.5%, ES 0.31, confidence limits 0.12;0.50) and mid-training (4.7%, ES 0.27, 0.12;0.43). Changes in PLA remained trivial. Thus, compared with PLA, IPC clearly increased power output in the first half in post- compared with pre- (6.1%, ES 0.30, −0.02;0.62) and mid-training (7.1%, ES 0.36, 0.06;0.65) and during the entire TT compared with mid-training (4.0%, ES 0.20, 0.06;0.35). As there was no change in any performance variables from pre- to mid-training, we focused our analysis of the physiological changes from pre- and mid- to post-training.

#### Physiological and Perceptual Responses

**Figure 3:**
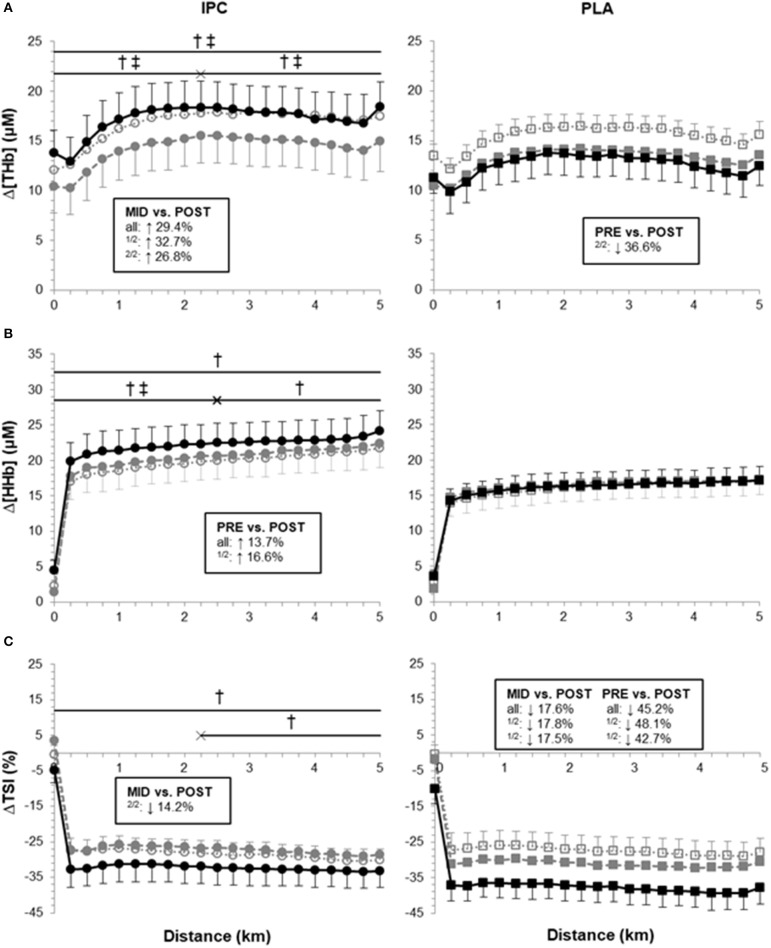
Average Δ[THb], Δ[HHb] and ΔTSI during the 5-km time trial at pre- (open symbols), mid- (gray symbols) and post-training (black symbols) in IPC and PLA. Data were as follows: **(A)** Average Δ[THb] for TT at pre- (IPC: 16.9 ± 2.9 μM; PLA: 15.5 ± 1.2 μM), mid- (IPC: 14.4 ± 2.8 μM; PLA: 13.3 ± 1.4 μM), and post-training (IPC: 17.4 ± 2.6 μM; PLA: 12.6 ± 2.1 μM). **(B)** Average Δ[HHb] for TT at pre- (IPC: 19.9 ± 2.6 μM; PLA: 16.1 ± 2.0 μM), mid- (IPC: 20.6 ± 2.5 μM; PLA: 16.5 ± 2.0 μM), and post-training (IPC: 22.3 ± 2.8 μM; PLA: 16.3 ± 1.9 μM). **(C)** Average ΔTSI for TT at pre- (IPC: −28.5 ± 3.3%; PLA: −27.5 ± 4.1%), mid- (IPC: −27.2 ± 3.4%; PLA: −31.2 ± 2.0%), and post-training (IPC: −32.4 ± 4.8%; PLA: −37.8 ± 4.3%). Within-group differences between time points are indicated in the square boxes for the first half (1/2), the second half (2/2) and the entire TT (all). (†) and (‡) indicate clear differences between-groups (IPC vs. PLA) at post- compared to pre- and mid-, respectively. Values are mean ± SE.

Muscle oxygenation variables are displayed in Figure 3. Overall, PLA did not alter Δ[HHb], but increased ΔTSI in post- compared with pre- (ES 0.78, 0.26;1.31) and mid-training (ES 0.52, −0.08;1.13). PLA also lowered Δ[THb] in the second half of the TT from pre- to post-training (ES −0.56, −1.37;0.25). On the other hand, IPC produced clear changes in all NIRS parameters. It increased Δ[THb] from mid- to post-training (ES 0.34, −0.02;0.69) which yielded clear differences with PLA in the first and second halves and the entire TT (post- vs. pre-: 73.6%, ES 0.70, −0.15;1.54, Figure 3A). From mid- to post-training, the increase in Δ[THb] was very largely correlated with the improvement in chronometric performance in IPC only (*r* = 0.77).

IPC also clearly increased Δ[HHb] in the first half of (ES 0.25, 0.18;0.32) and during the entire TT (ES 0.21, 0.14;0.27). Compared with PLA, IPC increased Δ[HHb] from pre- to post-training in the first (13.9%, ES 0.26, −0.07;0.59) and second half (11.7%, ES 0.22, −0.15;0.59), and during the entire TT (12.7%, ES 0.24, −0.11;0.59, Figure 3B). The increase in power output in the second half of the TT from pre- to post-training in IPC was very largely correlated with both Δ[HHb] (*r* = 0.71) and Δ[THb] (*r* = 0.71).

IPC increased ΔTSI (i.e., lowered absolute TSI) in the second half of the TT from mid- to post-training (ES 0.27, −0.21;0.74). Compared with PLA, IPC maintained ΔTSI during the entire TT from pre- to post-training (−24.2%, ES −0.57, −1.32;0.17, Figure 3C).

HR and S_p_O_2_ are displayed in Table 2. PLA did not alter S_p_O_2_, but increased HR from pre- to post-training. IPC decreased S_p_O_2_ in the second half and increased HR in post-, compared with pre- and mid-training during the entire TT. The increase in power output in the second half of the TT from pre- to post-training in IPC was correlated with HR (*r* = 0.73). Compared with PLA, IPC increased S_p_O_2_ and HR in post-, compared with pre- and mid-training.

**Table 2:** Average HR and S_p_O_2_ during the 5-km cycling time trial after IPC and PLA at pre-, mid-, and post-training.

	**IPC**			**PLA**			**ΔIPC vs**. **ΔPLA**	
	**PRE**	**MID**	**POST**	**PRE**	**MID**	**POST**	**MID vs. POST**	**PRE vs. POST**
	**Mean** **±** **SE**			**Mean** **±** **SE**			**%D (ES) CL**	
**Heart rate (beats per minute)**								
0.5 km	159 ± 4	160 ± 4	165 ± 4^ab^	158 ± 3	153 ± 5	160 ± 2^ab^	−2.4% (−0.17) −0.62;0.29	2.6% (0.17) −0.02;0.36
1.0 km	164 ± 4	164 ± 4	169 ± 4^ab^	163 ± 3	163 ± 3	165 ± 2	1.9% (0.13) −0.10;0.36	2.6% (0.17) −0.05;0.39
2.0 km	170 ± 3	169 ± 4	170 ± 4	168 ± 2	166 ± 2	169 ± 2^b^	−0.5% (−0.03) −0.17;0.11	0.3% (0.02) −0.17;0.21
3.0 km	173 ± 4	172 ± 4	174 ± 4	170 ± 2	169 ± 2	171 ± 2	1.1% (0.18) −0.22;0.59	0.6% (0.10) −0.38;0.57
4.0 km	175 ± 4	174 ± 4	178 ± 4^ab^	172 ± 2	172 ± 2	174 ± 2	1.6% (0.26) −0.09;0.61	1.4% (0.23) −0.12;0.58
5.0 km	180 ± 3	185 ± 4	188 ± 2^ab^	178 ± 2	180 ± 2	180 ± 2	**2.5% (0.42)** **−0.14;0.99**	**3.5% (0.59)** **0.08;1.10**
**Arterial O**_**2**_ **saturation (%)**								
0.5 km	96.8 ± 0.5	97.0 ± 0.6	97.5 ± 0.5	96.7 ± 1.4	98.4 ± 0.7	96.0 ± 1.5^b^	**1.4% (0.46)** **−0.10;1.02**	**1.4% (0.38)** **−0.03;0.79**
1.0 km	97.5 ± 0.4	97.1 ± 0.5	97.4 ± 0.6	96.6 ± 1.4	97.9 ± 0.6	96.6 ± 1.3	0.4% (0.12) −0.37;0.61	−0.2% (−0.06) −0.47;0.35
2.0 km	97.0 ± 0.3	96.2 ± 0.5	96.5 ± 0.5	96.3 ± 0.7	96.4 ± 0.6	96.4 ± 0.5	0.2% (0.06) −0.23;0.36	−0.7% (−0.19) −0.48;0.09
3.0 km	96.3 ± 0.3	95.7 ± 0.3	95.7 ± 0.4	95.8 ± 0.7	96.1 ± 0.7	95.8 ± 0.6	0.3% (0.20) −0.47;0.88	−0.6% (−0.33) −0.83;0.17
4.0 km	95.8 ± 0.4	95.3 ± 0.4	95.1 ± 0.5	95.8 ± 0.4	95.8 ± 0.5	95.6 ± 0.4	0.3% (0.21) −0.37;0.78	−0.1% (−0.05) −0.50;0.40
5.0 km	95.4 ± 0.4	94.8 ± 0.8	93.8 ± 0.8^a^	95.8 ± 0.7	94.8 ± 0.7	94.8 ± 0.5	−0.4% (−0.24) −1.04;0.55	0.1% (0.06) −0.80;0.93

*CL, confidence limits; %D, percentage difference between changes in IPC and PLA; ES, effect size*.

a,b *clear changes within each group in post- compared to pre- and mid-training, respectively*.

*Differences between changes in IPC and PLA are indicated in bold*.

RPE increased in both groups during the entire TT in post-, compared with pre- (PLA: 10.4%, ES 0.63, 0.26;1.00, IPC: 19.8%, ES 0.87, 0.39;1.34) and mid-training (PLA: 5.7%, ES 0.39, 0.10;0.67, IPC: 11.1%, ES 0.39, 0.10;0.67), and scores were higher in IPC –PLA (ES 0.31, −0.11;0.74).

### Maximal Incremental Step Test

Peak values at the end of exercise during maximal incremental step test and percentage differences between groups, from pre- to post-training, are displayed in Table 3. At maximal exercise, there was no change within and between groups for MAP, VO_2_, respiratory rate, S_p_O_2_, RPE, Δ[HHb], and Δ[THb]. However, IPC decreased respiratory exchange ratio and VCO_2_ at maximal exercise compared to PLA.

**Table 3:** Mechanical and physiological variables at the end of exercise during the maximal incremental step test after IPC and PLA at pre- and post-training.

	**IPC**		**PLA**		**ΔIPC VS. ΔPLA**
	**PRE**	**POST %d (ES)**	**PRE**	**POST %d (ES)**	**%D (ES) CL**
MAP (W)	359 ± 16	367 ± 15 2.3% (0.15)	360 ± 21	363 ± 19 1.3% (0.07)	1.0% (0.06) −0.19;0.32
VO_2_ peak (mL/min/kg)	57.6 ± 3.0	58.6 ± 2.7 2.1% (0.11)	58.1 ± 2.8	58.5 ± 2.4 1.0% (0.07)	1.1% (0.07) −0.29;0.43
VO_2_ peak (mL/min)	4341 ± 207	4427 ± 196 2.1% (0.13)	4308 ± 252	4351± 260 1.0% (0.05)	1.1% (0.07) −0.27;0.40
VCO_2_peak (mL/min)	4922 ± 138	4873 ± 153 −1.1% (0.10)	4691 ± 300	4832 ± 284 3.3% (0.16)	**−4.2% (−0.30)** **−0.61;0.02**
RERpeak	1.15 ± 0.04	1.11 ± 0.04 −3.2% (−0.27)	1.09 ± 0.04	1.12 ± 0.04 2.2% (0.19)	**−5.3% (−0.48)** **−1.08;0.11**
RRpeak (L/min)	163 ± 7	160 ± 6 −1.5% (−0.11)	165 ± 10	164 ± 7 0.1% (0.00)	−1.5% (−0.10) −0.78;0.58
HRpeak (bpm)	180 ± 3.7	179 ± 4.4 −0.5% (−0.07)	178 ± 3.6	175 ± 3.4^a^ −1.4% (−0.20)	0.8% (0.11) −0.21;0.43
S_p_O_2_ peak (%)	96.6 ± 0.3	96.1 ± 0.4 −0.6% (−0.43)	96.7 ± 0.6	96.6 ± 0.7 −0.1% (−0.06)	−0.4% (−0.27) −1.11;0.57
Δ[HHb]peak (μM)	21.2 ± 2.8	21.9 ± 2.0 8.0% (0.16)	15.3 ± 2.0	15.7 ± 1.9 2.1% (0.05)	5.8% (0.12) −0.42;0.67
Δ[THb]peak (μM)	12.7 ± 2.8	10.2 ± 1.2 −19.8% (−0.35)	8.3 ± 1.7	7.0 ± 1.7 −15.5% (−0.23)	−4.3% (−0.20) −1.10;0.71
ΔTSIpeak (%)	37.2 ± 4.3	34.6 ± 3.4 −1.4% (−0.03)	26.6 ± 2.1	33.1 ± 3.1^a^ 21.3% (0.57)	−18.8% (−0.49) −1.33;0.35

*CL, confidence limits; %d, in-group percentage difference between pre- and post-training; %D, percentage difference between changes in IPC and PLA; ES, effect size; Δ[HHb], change in deoxy-hemoglobin; HR, heart rate; MAP, maximal aerobic power; RER, respiratory exchange ratio; RR, respiratory rate; S_p_O_2_, arterial O_2_ saturation; Δ[THb], change in total hemoglobin; ΔTSI, change in tissue saturation index; VO_2_, O_2_ consumption; VCO_2_, carbon dioxide production*.

a *clear change within each group in post- compared to pre-training*.

*Differences between changes in IPC and PLA are indicated in bold. Values are mean ± SE*.

Mechanical and physiological variables at the respiratory compensation point during maximal incremental step test and percentage differences between groups, from pre- to post-training, are displayed in Table 4. Compared to PLA, IPC increased power output, VO_2_ and VCO_2_.

**Table 4:** Mechanical and physiological variables at the respiratory compensation point during the maximal incremental step test after IPC and PLA at pre- and post-training.

	**IPC**		**PLA**		**ΔIPC VS. ΔPLA**
	**PRE**	**POST %d (ES)**	**PRE**	**POST %d (ES)**	**%D (ES) CL**
Power (W)	291 ± 13	305 ± 13^a^ 4.7% (0.30)	303 ± 20	303 ± 17 0.6% (0.03)	**4.1% (0.24)** **−0.12;0.61**
%MAP	81.1 ± 1.4	83.7 ± 1.7^a^ 3.1% (0.44)	84.1 ± 1.7	83.6 ± 2.3 −0.8% (−0.09)	3.9% (0.54) −0.42;1.49
VO_2_ (mL/min)	3443 ± 187	3669 ± 178^a^ 6.8% (0.37)	3581 ± 256	3571 ± 219 0.3% (0.01)	**6.5% (0.33)** **0.00;0.67**
%VO_2_peak	79.22 ± 1.5	82.9 ± 1.5^a^ 4.6% (0.66)	82.6 ± 1.3	82.1 ± 1.5 −0.7% (−0.12)	**5.3% (0.85)** **−0.07;1.76**
VCO_2_ (mL/min)	3562 ± 120	3808 ± 143^a^ 6.8% (0.52)	3622 ± 241	3755 ± 221^a^ 4.1% (0.20)	2.6% (0.16) −0.35;0.67
%VCO_2_peak	72.5 ± 1.9	78.3 ± 2.3^a^ 8.0% (0.75)	77.2 ± 1.6	78.0 ± 2.5 0.9% (0.10)	**7.1% (0.73)** **−0.11;1.58**
RER	1.05 ± 0.03	1.05 ± 0.03 0.1% (0.01)	1.02 ± 0.03	1.06 ± 0.03^a^ 3.8% (0.42)	−3.6% (−0.39) −1.04;0.26

*CL, confidence limits; %d, in-group percentage difference between pre- and post-training; %D, percentage difference between changes in IPC and PLA; ES, effect size; MAP, maximal aerobic power; RER, respiratory exchange ratio; VO_2_, O_2_ consumption; VCO_2_, carbon dioxide production*.

a *clear change within each group in post- compared to pre-training*.

*Differences between changes in IPC and PLA are indicated in bold. Values are mean ± SE*.

### Blood Markers of Hypoxic Response and Blood Profile

Both IPC and PLA led to similar decreases in VEGF-α over time, but did not modify NO and HIF-1α. There was no difference between groups for all these markers (Table 5).

**Table 5:** Blood markers of the hypoxic response and blood profile after IPC and PLA at pre- and post-training.

	**IPC**		**PLA**		**ΔIPC VS. ΔPLA**
	**PRE**	**POST %d (ES)**	**PRE**	**POST %d (ES)**	**%D (ES) CL**
**Hypoxic response**					
HIF-1α (pg/mL)	2.48 ± 1.71	2.77 ± 1.94 11.7% (0.04)	1.69 ± 0.93	1.83 ± 1.09 7.8% (0.04)	3.9% (0.03) −0.09;0.16
VEGF-α (pg/mL)	23.2 ± 3.3	19.8 ± 3.8^a^ −19.5% (−0.37)	24.2 ± 4.6	19.2 ± 4.0^a^ −21.9% (−0.38)	3.1% (0.05) −0.47;0.58
NO (pg/mL)	40.1 ± 3.6	36.2 ± 2.1 −7.4% (−0.26)	43.2 ± 5.9	39.4 ± 3.4 −5.3% (−0.15)	−2.2% (−0.07) −0.87;0.73
**Blood profile**					
Erythrocytes (10^12^/L)	4.89 ± 0.10	4.92 ± 0.10 0.7% (0.10)	4.71 ± 0.11	4.70 ± 0.12 −0.2% (−0.02)	0.9% (0.12) −0.20;0.43
MCV (fL)	89.3 ± 0.9	89.2 ± 0.9 −0.2% (−0.06)	90.0 ± 0.8	89.2 ± 1.3 −1.0% (−0.25)	0.8% (0.22) −0.35;0.79
DIE (%)	13.2 ± 0.1	13.3 ± 0.2^a^ 1.0% (0.28)	13.3 ± 0.1	13.2 ± 0.1 −0.7% (−0.25)	**1.8% (1.05)** **−0.07;2.16**
Hemoglobin (g/L)	147 ± 2	148 ± 2 0.1% (0.01)	143 ± 2	141 ± 2 −0.9% (−0.18)	1.0% (0.19) −0.25;0.63
MCHC (pg)	30.2 ± 0.4	30.0 ± 0.4^a^ −0.9% (−0.20)	30.3 ± 0.3	30.1 ± 0.5 −0.9% (−0.19)	0.0% (0.00) −0.71;0.70
AHC (g/L)	338 ± 2	336 ± 2^a^ −0.7% (−0.42)	337 ± 2	338 ± 2 0.2% (0.09)	−0.9% (−0.52) −1.47;0.43
Ferritin (μg/L)	121 ± 21	128 ± 27 1.2% (0.02)	92 ± 18	82 ± 20^a^ −19.4% (−0.29)	**25.6% (0.32)** **−0.14;0.79**
Platelets (10^9^/L)	190 ± 11	200 ± 11^a^ 5.2% (0.24)	200 ± 10	194 ± 9 −2.7% (−0.17)	**8.1% (0.44)** **−0.02;0.89**
APV (fL)	9.69 ± 0.48	9.65 ± 0.43 −0.2% (−0.01)	8.58 ± 0.27	8.39 ± 0.24 −2.1% (−0.21)	1.9% (0.14) −0.19;0.47
Leucocytes (10^9^/L)	4.99 ± 0.36	5.40 ± 0.33^a^ 8.6% (0.37)	5.66 ± 0.61	4.91 ± 0.33^a^ −11.1% (−0.41)	**22.1% (0.84)** **0.06;1.62**
Neutrophils (%)	48.4 ± 1.7	51.0 ± 1.9^a^ 5.1% (0.39)	54.5 ± 1.8	53.7 ± 2.1 −1.8% (−0.15)	**7.0% (0.54)** **−0.01;1.09**
Eosinophils (%)	3.15 ± 0.57	2.65 ± 0.50^a^ −18.7% (−0.26)	2.47 ± 0.52	2.41 ± 0.44 1.3% (0.02)	**−19.7% (−0.33)** **−0.63;−0.03**
Basophils (%)	0.609 ± 0.039	0.609 ± 0.031 0.6% (0.03)	0.500 ± 0.047	0.533 ± 0.090 −2.6% (−0.05)	3.3% (0.09) −0.96;1.14
Lymphocytes (%)	39.2 ± 1.5	37.8 ± 1.9^a^ −4.2% (−0.25)	35.5 ± 1.6	36.3 ± 2.0 1.8% (0.11)	−5.9% (−0.37) −0.96;0.22
Monocytes (%)	8.57 ± 0.64	7.98 ± 0.45^a^ −5.8% (−0.24)	7.02 ± 0.46	7.10 ± 0.41 1.6% (0.08)	−7.3% (−0.33) −0.99;0.33

*AHC, average hemoglobin concentration; APV, average platelet volume; CL, confidence limits; %d, in-group percentage difference between pre- and post-training; %D, percentage difference between changes in IPC and PLA; DIE, distribute index of erythrocytes; ES, effect size; HIF-1α, hypoxia inducible factor-1α; MCHC, mean corpuscular hemoglobin content; MCV, mean corpuscular volume; NO, nitric oxide; VEGF-α, vascular endothelial growth-factor*.

a *clear change within each group in post- compared to pre-training*.

*Differences between changes in IPC and PLA are indicated in bold. Values are mean ± SE*.

The blood profile is summarized in Table 5. IPC increased platelets concentration and the distribute index of erythrocytes, and decreased mean corpuscular hemoglobin level and average hemoglobin concentration. PLA decreased hematocrit, ferritin, and platelets concentration post-training. There was a clear difference between groups for platelets, the distribute index of erythrocytes, hematocrit, and ferritin concentrations. Regarding the immune function, IPC increased total leucocytes and neutrophils, and decreased eosinophils, lymphocytes, and monocytes, while PLA decreased total leucocytes. Compared to PLA, IPC induced greater changes in leucocytes, neutrophils, monocytes, and eosinophils from pre- to post-training. However, all values and changes within and between groups remained within the normal clinical range of all variables for a healthy population.

The lipid-lipoprotein profile and glucose-insulin homeostasis are summarized in Table 6. IPC increased TG levels, whereas PLA increased TG and decreased free-fatty acid levels. There was a clear difference between-groups for LDL-cholesterol and free-fatty acid concentrations. Finally, IPC increased insulin levels, while PLA increased fasting glucose levels, and this yielded a clear difference between groups for fasting glucose levels. Importantly, all values and changes within and between groups were within the normal clinical range of all variables for a healthy population.

**Table 6:** Lipid-lipoprotein profile and glucose-insulin homeostasis after IPC and PLA at pre- and post-training.

	**IPC**		**PLA**		**ΔIPC VS. ΔPLA**
	**PRE**	**POST %d (ES)**	**PRE**	**POST %d (ES)**	**%D (ES) CL**
**Lipid-lipoprotein profile**					
Total CHOL (mmol/L)	4.18 ± 0.30	4.37 ± 0.34 4.2% (0.15)	4.01 ± 0.26	4.03 ± 0.24 0.8% (0.04)	3.3% (0.14) −0.18;0.45
Triglycerides (mmol/L)	0.900 ± 0.209	0.997 ± 0.200^a^ 15.1% (0.22)	0.80 ± 0.11	0.93 ± 0.10^a^ 19.3% (0.44)	−3.5% (−0.07) −0.57;0.43
HDL-CHOL (mmol/L)	1.51 ± 0.11	1.53 ± 0.10 1.7% (0.07)	1.52 ± 0.11	1.52 ± 0.08 1.3% (0.06)	0.5% (0.02) −0.43;0.47
LDL-CHOL (mmol/L)	2.25 ± 0.28	2.38 ± 0.28 7.5% (0.12)	2.12 ± 0.20	2.08 ± 0.19 −1.9% (0.06)	**9.6% (0.20)** **−0.08;0.49**
FFA (mmol/L)	0.078 ± 0.019	0.086 ± 0.021 15.5% (0.15)	0.107 ± 0.039	0.091 ± 0.057^a^ −44.1% (−0.56)	**106.5% (0.77)** **0.26;1.27**
**Glucose-insulin homeostasis**					
Glucose (mmol/L)	4.98 ± 0.09	5.05 ± 0.09 1.3% (0.19)	4.84 ± 0.12	5.13 ± 0.12^a^ 6.0% (0.74)	**−4.5% (−0.31)** **−0.59;−0.02**
Insulin (pmol/L)	35.8 ± 4.8	40.7 ± 5.6^a^ 14.3% (0.29)	30.7 ± 2.7	32.7 ± 1.7 9.4% (0.34)	4.5% (0.12) −0.59;0.83

*CHOL, cholesterol; CL, confidence limits; %d, in-group percentage difference between pre- and post-training; %D, percentage difference between changes in IPC and PLA; ES, effect size; FFA, free fatty acids; HDL, high-density lipoprotein; LDL, low-density lipoprotein*.

a *denote clear change within each group in post- compared to pre-training*.

*Differences between changes in IPC and PLA are indicated in bold*.

*Values are mean ± SE*.

## Discussion

This study investigated the potential of IPC to enhance performance adaptations following SIT and attempted to elucidate some potential physiological underpins. The main findings were that in endurance athletes, when compared to SIT alone, eight sessions of SIT immediately preceded by three cycles of bilateral occlusions led to greater improvements in mean power output (~5%) and completion time (~2%) during a 5-km maximal cycling time trial, as well as increased fatigue resistance (~8.5%) during a Wingate test. Peak power output during the Wingate test was increased similarly in both groups. Although the adaptive responses to both aerobic and anaerobic performances are complex, the current study presents some evidence to suggest that increased muscle perfusion and peripheral O_2_ extraction occurred after IPC.

Few studies have investigated performance changes after chronic IPC, and only one applied this manoeuver before a training stimulus. Four cycles of upper-limb occlusion repeated over a period of 7 to 9 days, without any training, had no effect (Banks et al., 2016) or increased VO_2_max (Lindsay et al., 2017). Lindsay et al. also reported an increase in MAP and Wingate performance in recreationally-active participants (Lindsay et al., 2017). In athletes, application of IPC alone would likely be insufficient to induce such improvements (Marocolo et al., 2018). Indeed, IPC applied once or twice a day for 7 consecutive days failed to improve 4-km TT in trained cyclists (Lindsay et al., 2018), but combining IPC with high-intensity training could trigger larger adaptations. Surprisingly, trained middle-distance runners did not improve VO_2_max or 1-km TT performance after 8 weeks of training alone or when preceded by IPC (Slysz and Burr, 2019). The absence of effect on VO_2_peak and MAP is confirmed in the present study, but we observed an improvement in TT performance. Two possibilities might explain the lack of impact of IPC in this earlier study. First, the use of unilateral occlusions may not have provided a sufficient stimulus to derive benefits from IPC (Kraus et al., 2014; Cocking et al., 2018). Second, the training was mostly composed of low intensity sessions and a few faster-pace runs, which was likely not sufficient to trigger adaptations in trained athletes. SIT, however, has been shown to improve endurance performance in trained cyclists (Laursen et al., 2002) and runners (Esfarjani and Laursen, 2007). Although time spent at >90% VO_2_max during SIT sessions is low (typically 0–60 s in trained cyclists for an entire training session), it has been suggested that muscle O_2_ demand is high, especially as the number of sprints increases, as suggested by low muscle oxygenation levels (Buchheit et al., 2012; Paquette et al., 2019). In the present study, both groups increased the peak power output during the Wingate test, but only the chronic application of bilateral IPC prior to SIT sessions improved TT performance in endurance athletes in just 4 weeks.

Of all performance tests realized in this study, the 5-km self-paced time trial would be the most relevant to athletes wishing to enhance their sport performances. Endurance performance is mainly determined by maximal cardiorespiratory fitness, exercise economy, and anaerobic capacity (Hawley et al., 1997). In our study, the maximal cardiorespiratory fitness and the anaerobic capacity do not seems to be involved. Indeed, the end variables in the incremental step test (VO_2_max and MAP), which is mainly determined by central components, were not improved and the Wingate test was increased similarly in both groups. On the other hand, the changes in muscle hemodynamics, visible only after 4 weeks and accompanying the endurance performance enhancement in both tests following IPC, seem to be a key factor. More precisely, local blood volume tended to decrease in both groups at mid- compared to pre-training, with no clear changes in muscle O_2_ extraction and, thus, on performance. However, after 2 more weeks with IPC, participants clearly improved their TT performance and the reduction of the local blood volume during exercise was reversed and returned to initial values despite the increase in power output. Coincident with this increase in Δ[THb] was an increase in Δ[HHb] and a better maintenance of ΔTSI, which likely reflects a matching between O_2_ extraction and delivery. The likely positive effect of muscle hemodynamics changes on performance after IPC is also confirmed by the correlation between Δ[THb] and chronometric performance, as well as Δ[HHb] and TT power output. In the PLA group, the Δ[THb] decreased throughout the 4 weeks and so did the ΔTSI, without any clear change in TT performance. It could be argued that the increase in HR could partly explain the increase in local blood volume and the performance considering the positive correlation between HR and power output. However, because the participants were free to adjust their workload, we cannot ascertain if the changes in cardiovascular hemodynamics are associated with IPC or to a higher work rate, the latter being the more plausible hypothesis. Indeed, acute IPC is mostly reported to have no effect on HR during maximal exercise (Bailey et al., 2012b; Kido et al., 2015; Salvador et al., 2016; Kilding et al., 2018). In addition, a difference in HR of ~1% can hardly explain alone the difference in local blood volume, especially since HR and Δ[THb] displayed different patterns. Therefore, we conclude from these results that IPC combined with SIT induces additional adaptations at the peripheral level by increasing muscle perfusion and O_2_ uptake during exercise which could, in turn, increase endurance performance.

The present results also indicated that endurance performance enhancement could be due to an improvement in ventilatory threshold. The observation of a greater respiratory compensation point, which is predominantly determined by peripheral mechanisms (Joyner and Coyle, 2008), and the decrease in VCO_2_ and respiratory exchange ratio at maximal exercise, suggest an improved oxidative capacity, visible only after IPC. These results, combined with the observed peripheral adaptations on muscle perfusion and O_2_ uptake, could suggest a speeding of the VO_2_ kinetics, which could reduce the O_2_ deficit and the reliance on substrate level phosphorylation. As mentioned earlier, very few studies have investigated the effects of chronic IPC on training, but studies on acute IPC confirmed this possibility. Indeed, acute IPC has been reported to speed HHb kinetics (Kido et al., 2015) and, without changing first ventilatory threshold, VO_2_peak and MAP, and to have a beneficial priming effect (i.e., a decrease in VO_2_ slow component amplitude in the heavy-intensity domain) on well-trained male cyclists (Kilding et al., 2018). Authors of this study suggested that this effect on VO_2_ kinetics could be responsible for the observed TT performance improvement (Kilding et al., 2018). This could also have been the case in our study considering the increase in muscle O_2_ extraction during the TT and the change in the respiratory compensation point. Taken together, these results suggested that the combination of IPC with SIT could have enhanced exercise efficiency by improving aerobic metabolism and could represent a perspective for subsequent studies. Investigations on metabolism during exercise could help answer some of the questions surrounding the effects of IPC resulting in changes in muscle oxygenation that are visible while exercising.

To date, most studies applying IPC chronically have reported promising adaptations, though they have mainly been done without exercise. Daily exposure to IPC for 7 days enhanced artery endothelial function and cutaneous vascular conductance, which persisted up to 1 week after the intervention (Jones et al., 2014). One-week exposure also increased skeletal muscle oxidative capacity (Jeffries et al., 2018). Longer application of IPC for 8 weeks also improved endothelial function, and this enhancement lasted another 2 weeks (Jones et al., 2015). These upregulated functions at rest might have taken place in the present study and explained, at least in part, some of the changes in [THb] and [HHb] during exercise. It has been suggested that shear stress and local tissue hypoxia from IPC could up-regulate HIF-1α, activate VEGF-α gene expression (Fukumura et al., 2001; Albrecht et al., 2013; Heusch et al., 2015), and increase levels of NO (Lochner et al., 2002). Our data do not reveal any chronic changes to these markers after training with IPC. This does not preclude their implication, as: (1) our methodology was limited to one blood sample post-training, and (2) we may have missed the window of transiently increased expression during adaptation in the weeks between the start of the training program and post-training blood sampling. For example, studies using an identical training intervention to ours, but using blood-flow restriction during the recovery periods after SIT, reported a greater expression of muscle HIF-1α 3 h after one session in trained individuals (Taylor et al., 2016a), but no change in skeletal muscle capillarity and mitochondrial protein content after a 4 week period (Mitchell et al., 2019). Another possibility is that the frequency of the IPC procedure (2x/week) was insufficient to induce molecular adaptations in these trained endurance athletes. For example, Kimura et al. applied IPC six times per day for 1 month and reported an increase in NO production and VEGF plasma concentration measured 14 h post IPC (Kimura et al., 2007), and most of the studies on repeated IPC for cardiac surgery applied IPC every day (Liang et al., 2015; Meng et al., 2015). Thus, the optimal dose or protocol to maximize the hypoxic and angiogenic signaling events following IPC remains unclear. Taken together, these data suggest that the ergogenic effects of IPC are mainly derived from transient functional changes (probably related to an improved vascular control) rather than structural modifications *per se*.

Finally, our results preclude a chronic role for a number of circulating factors in contributing to improved performance after repeated IPC application. Indeed, neither training with or without IPC chronically modified blood markers of hypoxic and angiogenic signaling or vasodilation, nor in factors related to blood O_2_ transport capacity measured 2 days post-training. The absence of changes in lipid-lipoprotein profile, fasting glucose, and insulin levels in athletes without dietary modification or weight issues is not surprising. Others have suggested that the immune system or inflammation could be of importance in adaption following IPC (Thijssen et al., 2016). For example, Czibik et al. reported that nuclear-factor-kappa B could be responsible for modified gene regulation after IPC (Czibik et al., 2008), and daily IPC for 10 days altered neutrophil function and leukocyte inflammatory gene expression in humans (Konstantinov et al., 2004; Shimizu et al., 2010). However, our data show that the repetition of IPC two times per week had no effect on immune system function, though we did not measure markers of inflammation or transient response immediately after training.

In conclusion, 4 weeks of bilateral IPC applied immediately before SIT sessions elicited greater gains in time-trial performance in endurance athletes than the same SIT prescription without IPC. This was mainly associated with enhanced local perfusion and muscle O_2_ extraction, while hematocrit, VO_2*max*_, and maximal aerobic power did not change. Furthermore, the present intervention seems to be both safe and sufficient to induce performance enhancements of interest to trained individuals.

## Data Availability Statement

All datasets generated for this study are included in the article/supplementary material.

## Ethics Statement

The studies involving human participants were reviewed and approved by Ethic committee from Laval University. The patients/participants provided their written informed consent to participate in this study.

## Author Contributions

PP-D, DJ, PM, and FB conceptualized and designed the research project. PP-D acquired the data and conducted the statistical analysis. PP-D interpreted results with assistance from DJ, PM, and FB. PP-D wrote the manuscript with revisions from DJ, PM, and FB. All authors reviewed and agreed upon the final manuscript.

## Conflict of Interest

The authors declare that the research was conducted in the absence of any commercial or financial relationships that could be construed as a potential conflict of interest.
